# Physician Weight Recommendations for Overweight and Obese Firefighters, United States, 2011–2012

**DOI:** 10.5888/pcd11.140091

**Published:** 2014-07-10

**Authors:** Michelle Lynn Wilkinson, Austin Lane Brown, Walker Seward Carlos Poston, Christopher Keith Haddock, Sara Anne Jahnke, Rena Sue Day

**Affiliations:** Michelle Lynn Wilkinson, Austin Lane Brown,The University of Texas Health Science Center at Houston, School of Public Health, Houston, Texas; Walker Seward Carlos Poston, Christopher Keith Haddock, Sara Anne Jahnke, National Development and Research Institutes, Leawood, Kansas.

## Abstract

**Introduction:**

National guidelines state that health care professionals (HCPs) should advise patients on the importance of maintaining a healthy weight. Firefighters have high rates of obesity, and cardiovascular events are the leading cause of line-of-duty deaths in firefighters. This study assessed the association of age and body mass index (BMI) with HCP weight recommendations among male firefighters.

**Methods:**

We used data on self-reported HCP weight recommendations and measured BMI from a 2011–2012 national sample of male firefighters (N = 1,002). HCP recommendations were recorded as no advice, maintain, gain, or lose weight, and BMI was categorized as normal (<25.0 kg/m^2^), overweight (25.0–29.9 kg/m^2^), class I obese (30.0–34.9 kg/m^2^), and class II or III obese (≥35.0 kg/m^2^). We used multinomial logistic regression to estimate the odds of receiving weight advice by age and BMI categories.

**Results:**

Most firefighters (96%) reported visiting an HCP in the past year. Most (69%) firefighters and 48% of class I to III obese firefighters reported receiving no weight advice. Higher BMI predicted HCP advice to lose weight (odds ratio class I obese vs normal weight: 12.98; 95% confidence interval: 5.38–31.34). Younger firefighters were less likely to receive weight loss advice than older firefighters, except among those who were class II or III obese.

**Conclusions:**

HCPs are important sources of health information for firefighters. Overweight and obese firefighters, particularly those who are younger, do not consistently receive HCP advice to lose weight. This marks a missed opportunity to prevent further weight gain and reduce obesity-related health outcomes.

## Introduction

More than 70% of US firefighters are overweight or obese (1), exceeding the national average for adults (2,3). Obesity is a major threat to firefighter health and safety and is associated with an increased risk of job-related disability (4,5), high blood pressure (2), and cardiovascular disease (6). National guidelines recommend that health care professionals (HCPs) consider the body mass index (BMI, weight in kilograms divided by the square of height in meters) of all patients and advise patients who are overweight (25.0–29.9 kg/m^2^) and obese (≥30.0 kg/m^2^) to reduce their weight (7,8). Guidelines also recommend that HCPs counsel people with obesity-related comorbidities on the importance of maintaining a healthy weight (7).

Evidence-based research demonstrates the benefit of HCP weight counseling in the general population. HCP advice to lose weight is associated with a patient’s desire to lose weight (9), attempts to lose weight (10), and successful weight loss (11) in adult populations. Despite the apparent benefits of HCP weight recommendations, the prevalence of overweight and obese individuals receiving weight advice remains low in the general population, ranging from 40% to 50% for obese (12–14) and 15% to 65% for overweight individuals (13,15). Furthermore, a substantial proportion of obese and overweight (9) patients are not diagnosed by HCPs as obese or overweight. Results from studies of the general population suggest that HCPs are more likely to advise patients with obesity-related comorbidities (14–16), who are older (14,16), and who have greater severity of obesity (BMI ≥35 kg/m^2^) to lose weight (12).

HCP advice on weight loss could affect the weight status of firefighters as it does for those in the general population. However, the prevalence of HCP weight loss advice has not been investigated in the fire service. The objective of this study was to describe the prevalence of HCP weight advice among male career firefighters and assess the association of age and BMI with receiving advice.

## Methods

This study includes data from the Fuel 2 Fight cohort study (2011–2012) (17), which assessed risk factors for obesity and cardiovascular disease and their associations with the health and safety of career firefighters. The institutional review boards of the National Development and Research Institutes, Inc, and The University of Texas Health Science Center at Houston reviewed and approved study protocols.

A detailed description of the sampling plan and measurements has been reported elsewhere (17). In brief, 10 fire departments implementing a wellness and fitness approach (WA) were matched to 10 departments of similar size, call volume, staffing, and catchment area that were not following the health promotion approaches (Standard). Fire departments were defined as following a WA if they implemented key pieces of the Fire Service Joint Labor Management Wellness-Fitness Initiative (18), which recommends annual medical evaluations and outlines how to start wellness and fitness programs, instructions for rehabilitation, and appropriate behavioral health interventions. The sample was firefighters recruited from 3 randomly selected stations in selected departments. For larger departments, 2 battalions or divisions were randomly selected and 3 stations within each were randomly selected for inclusion. The participation rate of firefighters attending the station study recruitment sessions was 94.4%.

Firefighters completed a self-administered general health assessment including the question, “In the past 12 months, has a doctor, nurse, or other health professional given you advice about your weight?” Among firefighters reporting an HCP visit in the past 12 months, responses were recorded as “no advice, advice to maintain weight, advice to gain weight, and advice to lose weight.” Additionally, each firefighter provided information on demographic and occupational (rank in department) status and substance use (smoking and alcohol consumption), and a medical history listing any comorbidities. Firefighters were specifically asked if they had any of the following conditions associated with obesity: type 1 diabetes, type 2 diabetes, high blood pressure, high cholesterol, arthritis, asthma, heart disease, stroke, emphysema, sleep apnea, celiac disease, lactose intolerance, lectin intolerance, and cancer. Questions on self-reported tobacco and alcohol use were adapted from previously validated instruments (19,20).

Height and weight were measured by trained investigators using a portable stadiometer and scale. BMI was calculated from height and weight measurements, and categories of BMI were defined by using standard cutpoints (7). Firefighters’ weight status was classified as normal weight (<25.0 kg/m^2^), overweight (25.0–29.9 kg/m^2^), class I obese (30.0–34.9 kg/m^2^), or class II or III obese (≥35.0 kg/m^2^). Body fat percentage (BF%) and waist circumference (WC) were measured by using standard protocols (7,17,21).

Statistical analyses were conducted by using Stata version 12.1 (StataCorp, LP, College Station, Texas). Descriptive statistics were generated as means and standard deviations or frequencies and proportions, where appropriate, by outcome categories. Female firefighters (n = 22) were excluded because of their small numbers, although these were consistent with national estimates (22). Multinomial logistic regression was used to determine the associations between HCP weight advice, age (mean-centered), and BMI, controlling for other covariates. Backward elimination and evaluation of the likelihood-ratio test led to the development of the most parsimonious model. Additional models were used to assess the effects of BF% and WC on receiving HCP weight advice. Among the Fuel 2 Fight firefighters, correlations between measures of adiposity were all greater than 0.80. To assess potential misclassification of overweight firefighters, a secondary analysis was restricted to those firefighters defined as both fit by BF% and normal by WC (BF% <18 and WC <94 cm) (7,21,28). 

## Results

Of the 1,002 male firefighters, 924 (92.2%) reported visiting an HCP within the past year (Table 1). Overall, 82.5% of firefighters were overweight (BMI of 25.0–29.9 kg/m^2^) or obese (BMI of ≥30.0 kg/m^2^). Most firefighters were nonminorities (65.4%), nontobacco users (72.3%), worked in WA departments (52.2%), and consumed alcohol, although 15% reported abstaining from alcohol in the past 30 days. Almost half reported at least 1 obesity-related comorbidity. 

**Table 1:** Demographic and Behavioral Characteristics of US Male Firefighters, Fuel 2 Fight, 2011–2012

Characteristic^a^	Total	Weight Advice in Past Year			
		No Advice	Maintain	Gain	Lose
	N = 924	N = 639	N = 73	N = 17	N = 195
**Age, mean (SD)**	39.3 (8.8)	38.1 (8.9)	39.8 (8.0)	42.9 (10.2)	42.6 (8.0)
**Rank^b^**					
Firefighter	616 (67.8)	441 (70.3)	42 (57.5)	12 (80.0)	121 (62.7)
Officer	199 (21.9)	131 (20.9)	20 (27.4)	1 (6.7)	47 (24.4)
Chief	38 (4.2)	20 (3.2)	5 (6.9)	2 (13.3)	11 (5.7)
Other	55 (6.1)	35 (5.6)	6 (8.2)	0	14 (7.3)
**Ethnicity^b^**					
Nonminority	594 (65.4)	426 (67.4)	47 (64.4)	10 (66.7)	111 (58.7)
Minority	315 (34.6)	206 (32.6)	26 (35.6)	5 (33.3)	78 (41.3)
**BMI^b^^,^ c**					
Normal weight	161 (17.5)	132 (20.7)	20 (28.2)	3 (17.7)	6 (3.1)
Overweight	487 (53.1)	374 (58.7)	46 (64.8)	9 (52.9)	58 (30.1)
Class I obese	201 (21.9)	109 (17.1)	5 (7.0)	3 (17.7)	84 (43.5)
Class II or III obese	69 (7.5)	22 (3.5)	0	2 (11.8)	45 (23.3)
**Comorbidities^d^**					
None	523 (55.6)	384 (60.9)	40 (58.3)	7 (41.2)	67 (34.4)
Any	417 (44.4)	247 (39.1)	30 (41.7)	10 (58.8)	128 (65.6)
**Tobacco use^b^^,^ d**					
None	682 (72.3)	450 (70.9)	51 (69.9)	11 (73.3)	153 (78.5)
Any	261 (27.7)	185 (29.1)	22 (30.1)	4 (26.7)	42 (21.5)
**Alcohol, drinks/d^b^^,^ e**					
Abstinent	137 (14.6)	91 (14.4)	7 (9.9)	1 (6.3)	36 (18.8)
1 or 2	381 (40.6)	249 (39.3)	32 (45.1)	5 (31.3)	82 (42.7)
3 or 4	260 (27.7)	179 (28.3)	20 (28.2)	6 (37.5)	44 (22.9)
≥5	161 (17.1)	114 (18.0)	12 (16.9)	4 (25.0)	30 (15.6)
**Department type**					
Standard	453 (47.8)	313 (49.0)	24 (32.9)	2 (11.8)	90 (46.2)
WA	495 (52.2)	326 (51.0)	49 (67.1)	15 (88.2)	105 (53.9)

Abbreviations: SD, standard deviation; BMI, body mass index; WA, wellness and fitness approach.

a Values are expressed as n (%) unless otherwise indicated.

b Missing: weight advice (37 had not been to the doctor in the past 12 months and 41 were missing); rank (n = 16); minority (n = 15); BMI (n = 6); comorbidities (n = 9); tobacco (n = 6); alcohol (n = 12).

c BMI categories: normal weight (<25.0 kg/m^2^), overweight (25.0–29.9 kg/m^2^), class I obese (30.0–34.9 kg/m^2^), and class II or III obese (≥35.0 kg/m^2^).

d Comorbidities were type 1 diabetes, type 2 diabetes, high blood pressure, high cholesterol, arthritis, asthma, heart disease, stroke, emphysema, sleep apnea, celiac disease, lactose intolerance, lectin intolerance, and cancer.

e Tobacco use in the past 30 days includes smoking cigarettes or cigars or using chewing tobacco.

Most firefighters (69.2%) reported receiving no weight advice in the past year, and fewer than half of the obese (BMI ≥30.0 kg/m^2^) firefighters reported being advised to lose weight. Among overweight firefighters, only 12% received advice to lose weight. On average, firefighters who did not receive weight advice were younger (mean age for no advice: 38.1 years vs any advice: 41.9 years), nonminorities (66.7%) with lower BMI (mean BMI for no advice: 27.7 kg/m^2^ vs any advice: 30.4 kg/m^2^) and less likely to report comorbidities (60.1%) than firefighters who reported receiving weight advice.

The proportion of firefighters reporting any weight recommendation (maintain, gain, or lose) versus no weight recommendation was determined for each combination of BMI and age category (Table 2). Fewer than half (46.8%) of firefighters who were class I obese and aged 40 to 49 years reported receiving any weight recommendation from a health professional. Conversely, of those who were class I obese and aged 20 to 29, only 13.0% reported receiving weight advice. In each age category, firefighters were more likely to receive weight advice with an increase in BMI classification. Likewise, the proportions of firefighters who reported receiving weight recommendations increased with age and BMI category. Overall, most (68.1%) class II and III obese firefighters reported receiving weight advice from an HCP, and the proportion reporting advice was consistent across age categories.

**Table 2:** Percentage of US Male Firefighters Receiving Any Health Professional Weight Advice by Age and Body Mass Index (BMI), Fuel 2 Fight, 2011–2012

Age, y	Body Mass Index (%)^a^			
	Normal Weight	Overweight	Class I Obese	Class II or III Obese
20–29	9.8	11.9	13.0	66.7
30–39	12.7	19.1	52.2	66.7
40–49	27.3	28.7	46.8	70.0
50–60	45.5	30.8	51.1	66.7

a BMI categories: normal weight (<25.0 kg/m^2^), overweight (25.0–29.9 kg/m^2^), class I obese (30.0–34.9 kg/m^2^), and class II or III obese (≥35.0 kg/m^2^).

Results from multinomial regression modeling appear in Table 3. The maintain and gain estimates were collapsed because they did not differ meaningfully and had small cell sizes. Firefighters reporting advice to maintain or gain weight were more likely to work in WA departments (odds ratio [OR] 2.19; 95% confidence interval [CI] 1.33–3.60) compared with firefighters reporting no advice. No other variables were significantly associated with receiving advice to maintain or gain weight. Conversely, belonging to a WA department (OR, 1.51; 95% CI, 1.03–2.21), having prevalent comorbidities (OR, 1.68; 95% CI, 1.14–2.47), being older, and having a higher BMI were all significant predictors of receiving advice to lose weight.

**Table 3:** Multinomial Logistic Regression of Health Care Professional Weight Advice to US Firefighters, Relative to No Advice, Fuel 2 Fight, 2011–2012

Characteristic	Advice to Maintain or Gain Weight		Advice to Lose Weight	
	OR (95% CI)	*P* Value^a^	OR (95% CI)	*P *Value^a^
**Body mass index^b^**				
Normal weight	1 [Reference]	NA	1 [Reference]	NA
Overweight	0.73 (0.42–1.26)	.26	2.75 (1.15–6.58)	.02
Class I obese	0.39 (0.16–0.92)	.03	12.98 (5.38–31.34)	<.001
Class II or III obese	0.58 (0.13–2.72)	.49	39.49 (14.64–106.49)	<.001
**Age, y^c^**	1.03 (1.00–1.06)	.04	1.04 (1.02–1.07)	<.001
**Comorbidities^d^**	1.24 (0.77–1.99)	.38	1.68 (1.14–2.47)	.009
**WA**	2.19 (1.33–3.60)	.002	1.51 (1.03–2.21)	.03

Abbreviations: OR, odds ratio; CI, confidence interval; NA, not applicable; WA, wellness and fitness approach.

a Calculated using the Wald χ^2^ test.

b BMI categories: normal weight (<25.0 kg/m^2^), overweight (25.0–29.9 kg/m^2^), class I obese (30.0–34.9 kg/m^2^), and class II or III obese (≥35.0 kg/m^2^).

c Mean-centered age.

d Comorbidities were type 1 diabetes, type 2 diabetes, high blood pressure, high cholesterol, arthritis, asthma, heart disease, stroke, emphysema, sleep apnea, celiac disease, lactose intolerance, lectin intolerance, and cancer.

Interaction terms between BMI categories and age were significant, suggesting that the effect of BMI category on receiving advice to lose weight depends on the age of the participant (Figure). Holding age constant, higher BMI categories were associated with higher odds of receiving advice to lose weight. For example, the odds of receiving advice to lose weight were 63.7 times greater for class II or III obese firefighters aged 45 relative to normal-weight firefighters of the same age. Likewise, the effect of age on receiving advice to lose weight depends on the BMI category of the individual. Each year increase in age for firefighters with normal BMI corresponds to a 21% increase in the odds of receiving advice to lose weight compared with receiving no advice. The youngest firefighters are less likely to receive weight loss advice than firefighters in older age groups. The odds of receiving weight loss advice for an obese firefighter aged 45 is 2.22 times the odds for an obese firefighter aged 25. As BMI categories increase, the effect of age on advice to lose weight becomes less pronounced. At the highest BMI category, the youngest firefighters are no more likely to receive weight loss advice than the older age groups (OR for interaction <1.0).

**Figure:**
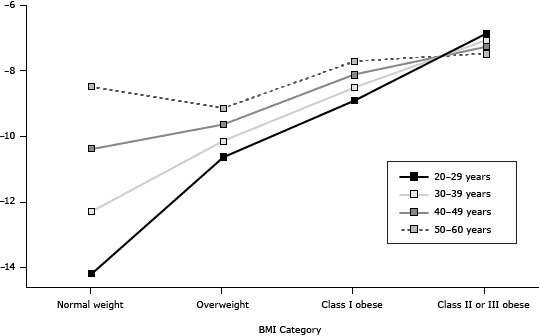
Probability of receiving health care professional weight loss advice, by body mass index (BMI) and age, among male US firefighters in the Fuel 2 Fight study, 2011–2012. Model with interaction: ln=−3.99+1.55(Overweight)+3.14(Class I Obese)+4.32(Class II-III Obese)+0.19(Age)–0.14(Overweight×Age)–0.15(Class I Obese×Age)–0.21(Class II-III Obese×Age)+0.53(Comorbidities)+0.41(WA). Log odds are based on the mean for each age category. **Table:** 

BMI Category/Age, y	Log Odds
Normal weight	
20–29	−14.16
30–39	−12.26
40–49	−10.36
50–60	−8.46
Overweight	
20–29	−10.61
30–39	−10.11
40–49	−9.61
50–60	−9.11
Class I obese	
20–29	−8.88
30–39	−8.48
40–49	−8.08
50–60	−7.68
Class II or III obese
20–29	−6.84
30–39	−7.04
40–49	−7.24
50–60	−7.44

Secondary analyses were conducted using WC and BF% in place of BMI. Higher measures of WC and BF% were both significantly associated with receipt of HCP weight loss advice. Interaction terms for age and WC and age and BF% were also significant at the *P* = .05 level. Because the results for WC and BF% were similar and BMI is the most common estimate of adiposity, only the BMI results are presented. An additional model was constructed for BMI excluding BMI-defined overweight firefighters with BF% <18% and WC <94cm. Exclusion of these firefighters did not meaningfully affect the results; thus, the results from the entire cohort are presented. 

## Discussion

In this national cohort of career firefighters, many did not report receiving weight advice from an HCP. As found in previous studies of US adults (12–14), less than half of obese (BMI ≥30.0 kg/m^2^) firefighters were advised to lose weight by an HCP. However, fewer overweight firefighters received weight loss counseling than reported estimates for the general population (13,15). This study found that comorbidities were predictive of receipt of weight loss advice, which is consistent with previous research (14–16). Still, only 30% of firefighters with an obesity-related comorbidity were advised to lose weight.

The results of this study suggest that receipt of HCP advice to lose weight depends on the joint effects of age and BMI. Young overweight and class I obese firefighters were less likely to receive advice to lose weight than their older counterparts. On the other hand, class II or III obese firefighters were more likely to receive weight loss advice, regardless of age, than those with lower BMI. The increase in the prevalence of weight loss advice observed with increasing age, BMI, and comorbidities indicates that many health care providers may reserve weight loss counseling for individuals with established obesity-related complications. Understandably, HCPs are more likely to counsel firefighters at highest risk for obesity-related adverse health outcomes; however, the scarcity of weight counseling in young overweight or class I obese firefighters marks a missed opportunity to thwart weight gain in younger firefighters, potentially delaying or preventing the onset of obesity-related morbidities. Young adults are at greater risk for weight gain than older adults, and overweight young adults are more likely than healthy weight young adults to experience additional weight gain (23,24). A 10-year longitudinal study found that young men aged 25 to 34 had a greater increase in BMI than older (≥35 years) men, and most adults gain weight during the transition from young adulthood to middle age (25). HCP weight counseling is essential to prevent and treat the complications of obesity (7,26) and must be provided to all overweight and obese firefighters regardless of age.

Firefighters from departments reporting implementation of Wellness-Fitness Initiative recommendations (WA departments) had higher odds of receiving any weight loss advice from an HCP than firefighters in Standard departments. The Wellness-Fitness Initiative is a voluntary program recommending annual medical evaluations; thus, participating departments encourage firefighters to be more aware of their health risks. Additionally, firefighters belonging to departments following the Wellness-Fitness Initiative may be more likely to see HCPs specializing in occupational medicine. Occupational medicine doctors may be more aware of the health risks associated with obesity in the fire service than primary care physicians, increasing the likelihood of weight counselling. In this study, firefighters were not asked what kind of HCP provided the advice (ie, a personal physician or the department doctor).

We found no association between tobacco and alcohol use and receipt of HCP weight loss advice. Regular tobacco use and excessive alcohol intake are known risk factors for cardiovascular disease and interact with weight status. Overweight and obese adults who either drink alcohol excessively or use tobacco regularly are at higher risk of adverse cardiovascular outcomes than nonusers of tobacco and moderate drinkers. Although tobacco and alcohol use were not significant in our model, tobacco use and higher levels of alcohol intake were associated with lower odds of receiving weight loss advice. Therefore, it is possible that HCPs are counseling firefighters on the health risks of tobacco and alcohol use instead of weight loss.

There are some limitations to consider. First, firefighters were asked to recall conversations with an HCP in the 12 months before participation in the study. Recall of advice by firefighters could have been influenced by the method or intensity of HCP advice delivery. It is possible that advice to lose weight was given to some firefighters but the impact of the message was insufficient to leave a lasting impression. Similarly, this study could not assess why HCPs did not recommend weight loss to overweight and obese firefighters. Previous research has found that HCPs lack confidence in their ability to diagnose and treat obesity (27). HCPs may feel unable to counsel firefighters properly on weight loss. Additionally, the temporal relationship between previous weight loss advice and BMI cannot be established. Conversations with an HCP occurred in the 12 months before participation in the study; thus, it is possible that BMI measured in this study differs from BMI at the time of the HCP visit.

This study primarily used BMI to define obesity instead of alternative estimates of adiposity. BMI does not distinguish between lean and fat mass and may not be an appropriate metric for defining overweight and obesity in populations with high levels of muscle mass (28). The proportion of overweight firefighters receiving weight loss advice increased slightly from the proportion of BMI-only defined overweight (12.75% and 12%, respectively). Our study and previous studies of firefighters demonstrate high agreement between BMI, WC, and BF% for defining obesity (29,30). Because BF% and WC assessments are not part of routine physical assessments for many HCPs and national recommendations for the diagnosis and treatment of obesity are based on BMI (7,26), BMI was used as the main measure of adiposity in this study.

This study does have many strengths; height and weight were measured by study staff as opposed to using self-reported height and weight, which is subject to bias (17). Furthermore, this is a large, ethnically diverse national sample of firefighters, including firefighters from US territories. This sample had a higher rate of ethnic minority firefighters than national rates for the fire service and a high participation rate. Thus, the results of this research may be generalizable to the greater US male career firefighter population.

Of note in this study was the finding that 10 firefighters with BMI of 30.0 kg/m^2^ or more were advised to maintain or gain weight by an HCP. These firefighters might have misunderstood the advice given to them, recalled it differently during data collection, or made a mistake when completing the questionnaire. Additionally, HCPs could have misdiagnosed these firefighters as not obese and given weight advice appropriate to nonobese persons. Of these firefighters, 9 were classified as obese by either body fat percentage or waist circumference and 1 was borderline obese according to BF%. Finally, this finding could be due to this study’s inability to determine temporality between advice and BMI. These 10 firefighters may have experienced weight change since seeing a HCP; thus, the advice given at the time may have been appropriate.

Firefighters are a high-risk group for obesity, which increases their risk for injury, disability, and mortality (2,6). Reducing overweight and obesity in the fire service could correspond to an improvement in overall firefighter health and public safety. HCPs should appropriately advise firefighters on weight loss and maintenance according to national guidelines (26); however, this study identified inconsistent weight loss advice for overweight and obese firefighters. In particular, young overweight and obese firefighters are not receiving adequate weight counseling. Previous studies of HCP advice in the general population have found a significant positive association with advice and successful attempts to lose weight (11). The lack of HCP intervention on overweight and obese firefighters’ weight marks a missed opportunity for prevention of obesity-related comorbidities in the fire service. Recently, the American Heart Association, American College of Cardiology, and The Obesity Society released the “2013 Guidelines for the Management of Overweight and Obesity in Adults” (26). This report provides evidence-based guidelines for HCPs and recommends that every adult patient be screened annually for overweight and obesity using BMI and WC standard cutpoints. Patients identified as overweight or obese should receive counseling on the risks for cardiovascular disease (CVD), type 2 diabetes, and all-cause mortality associated with increased adiposity and prescribed dietary strategies for weight loss and lifestyle interventions, as necessary (26). Additional research is necessary to evaluate the effect of the updated guidelines on HCP weight counseling practices. However, these guidelines provide the framework necessary to improve physician management of overweight and obesity. Future research should investigate barriers to receiving weight loss advice and health benefits of HCP weight loss counseling in the fire service.
